# The association of dimethylarginine dimethylaminohydrolase 1 gene polymorphism with type 2 diabetes: a cohort study

**DOI:** 10.1186/1475-2840-10-16

**Published:** 2011-02-09

**Authors:** Tse-Min Lu, Shing-Jong Lin, Ming-Wei Lin, Chiao-Po Hsu, Ming-Yi Chung

**Affiliations:** 1 Division of Cardiology, Department of Internal Medicine, Taipei Veterans General Hospital, Taipei, Taiwan, R.O.C; 2 School of Medicine, National Yang-Ming University, Taipei, Taiwan, R.O.C; 3 Department of Medical Research and Education, Taipei Veterans General Hospital, Taipei, Taiwan, R.O.C; 4 Institute of Public Health, National Yang-Ming University, School of Medicine, Taipei, Taiwan, R.O.C; 5 Division of Cardiovascular Surgery, Department of Surgery, Taipei Veterans General Hospital, Taipei, Taiwan, R.O.C; 6 Department of Life Sciences and Institute of Genome Sciences, National Yang-Ming University, Taipei, Taiwan, R.O.C

## Abstract

**Background:**

Elevated plasma levels of asymmetric dimethylarginine (ADMA) has been reported to be associated with insulin resistance and micro/macrovascular diabetic complications, and may predict cardiovascular events in type 2 diabetic patients. Dimethylarginine dimethylaminohydrolase 1 (DDAH1) is the major enzyme eliminating ADMA in humans, but the effect of genetic variations in *DDAH1 *on type 2 diabetes and its long-term outcome are unknown.

**Methods:**

From July 2006 to June 2009, we assessed the association between polymorphisms in *DDAH1 *and type 2 diabetes in 814 consecutive unrelated subjects, including 309 type 2 diabetic patients and 505 non-diabetic individuals. Six single nucleotide polymorphisms (SNPs) in *DDAH1*, rs233112, rs1498373, rs1498374, rs587843, rs1403956, and rs1241321 were analyzed. Plasma ADMA levels were determined by high performance liquid chromatography. Insulin sensitivity was assessed by the homeostasis model assessment of insulin resistance (HOMA-IR).

**Results:**

Among the 6 SNPs, only rs1241321 was significantly associated with a decreased risk of type 2 diabetes (AA *vs *GG+AG, OR = 0.64, 95% CI 0.47-0.86, p = 0.004). The association remained unchanged after adjustment for plasma ADMA level. The fasting plasma glucose and log HOMA-IR tended to be lower in subjects carrying the homozygous AA genotype of rs1241321 compared with the GG+AG genotypes. Over a median follow-up period of 28.2 months, there were 44 all-cause mortality and 50 major adverse cardiovascular events (MACE, including cardiovascular death, non-fatal myocardial infarction and stroke). Compared with the GG and AG genotypes, the AA genotype of rs1241321 was associated with reduced risk of MACE (HR = 0.31, 95% CI: 0.11-0.90, p = 0.03) and all-cause mortality (HR = 0.18, 95% CI: 0.04-0.80, p = 0.02) only in subgroup with type 2 diabetes. One common haplotype (GGCAGC) was found to be significantly associated with a decreased risk of type 2 diabetes (OR = 0.67, 95% CI = 0.46-0.98, p = 0.04).

**Conclusions:**

Our results provide the first evidence that SNP rs1241321 in *DDAH1 *is associated with type 2 diabetes and its long-term outcome.

## Introduction

Endothelial dysfunction is present from the onset of type 2 diabetes and strongly related to its outcomes [1], and itself may lead to the development of insulin resistance and diabetes [2,3]. Derangement of the L-arginine-nitric oxide (NO) pathway by asymmetric dimethylarginine (ADMA) has been implicated as an important contributing factor for endothelial dysfunction. ADMA is characterized as a circulating endogenous inhibitor of NO synthase by competing with L-arginine as the substrate [4,5]. Moreover, ADMA may increase oxidative stress by uncoupling the electron transport between NO synthase and L-arginine, which can lead to decrease in the production and availability of endothelium-derived NO [6,7]. Elevated plasma ADMA levels have been observed in patients with various risk factors for atherosclerosis, including insulin resistance and type 1/2 diabetes [8-10], and have been reported to be associated with micro/macrovascular diabetic complications [11,12]. Moreover, several prospective studies have demonstrated that elevated plasma ADMA level may predict adverse cardiovascular events in type 2 diabetic patients [13,14]. Taken together, these evidences suggest that ADMA may be a novel pathogenic factor of diabetes.

In the human body, 80% of ADMA is metabolized to citrulline by dimethylarginine dimethylaminohydrolase (DDAH) and the remainder is excreted by the kidneys [15]. There are 2 isoforms of DDAH, with DDAH1 present mainly in the kidneys and brain, while DDAH2 is predominantly found in the heart and kidney [16]. Mice over-expressing *DDAH1 *have lower plasma ADMA levels and are insulin-sensitive, presumably by reducing glucose uptake by skeletal muscle or by diminishing insulin signaling in the liver [17]. However, the effect of genetic variations of *DDAH1 *on type 2 diabetes has not been reported. In this prospective study, we assessed the association between single nucleotide polymorphisms (SNPs) in *DDAH1 *and type 2 diabetes and their long-term outcome in a population undergoing diagnostic coronary angiography.

## Materials and methods

### Study population

From July 2006 to June 2009, 814 consecutive unrelated subjects, including 309 type 2 diabetic patients and 505 non-diabetic control subjects, were recruited at Taipei Veterans General Hospital from the patients scheduled to undergo coronary angiography for chest pain and/or suspected coronary artery disease. Exclusion criteria included patients with severe liver disease and end-stage renal disease, active infectious disease, chronic or acute inflammatory disease, malignancy, and unstable hemodynamic status. Thorough medical histories of all participants were recorded. Type 2 diabetes mellitus was diagnosed according to the diagnostic criteria defined by the American Diabetes Association in 2003 [18] or when the individual was receiving oral hypoglycemic agents or insulin injection therapy at the time of recruitment. Most of the diabetic patients have medication with oral hypoglycemic agents (n = 244, 79.0%, including metformin, n = 185, 60.0%, sulfonylurea, n = 94, 30.4%, thiazolidinedione, n = 10, 3.2%, acarbose, n = 30, 9.7%), or insulin alone (n = 49, 15.8%), or insulin combined with oral hypoglycemic agents (n = 8, 2.6%), while a few received only dietary therapy only (n = 8, 2.6%). Hypertension was diagnosed according to the Seventh Joint National Committee criteria [19] or if the patient was receiving anti-hypertensive drugs. All medications, cigarette smoking and beverages containing alcohol or caffeine were withdrawn for at least 12 hours. Fasting blood samples were collected in EDTA tubes for the measurement of ADMA, blood sugar, insulin, and for other biochemical and genotype analyses before the coronary angiography in the morning. After coronary angiography, all patients with ≥50% stenosis in at least one major coronary artery underwent successful revascularization procedures, including percutaneous coronary intervention or coronary artery bypass surgery if indicated. All patients were prospectively followed by monthly office visit or by telephone contact and chart review for the occurrence of all-cause mortality and first-ever major adverse cardiovascular events (MACE), defined as cardiovascular death, stroke and non-fatal myocardial infarction. Cardiovascular death was diagnosed as any death with definite cardiovascular cause or any death that was not clearly attributed to a non-cardiovascular cause. Myocardial infarction was defined as the presence of significant new Q waves in at least 2 electrocardiographic leads or of symptoms compatible with myocardial infarction associated with increase in creatine kinase-MB fraction ≥3 times the upper limit of the reference range. The study protocol was approved by the Institutional Review Board at Taipei-Veterans General Hospital and all participants provided written informed consent.

### Laboratory measurements

Blood samples were centrifuged at 3000 rpm for 10 minutes immediately after collection and plasma was frozen until analysis. Plasma L-arginine and ADMA concentrations were determined by high performance liquid chromatography as described previously [20]. The recovery rate for ADMA was > 90%, and the within-assay and between-assay variation coefficients were not more than 7% and 8%, respectively. Fasting serum creatinine, total and high-density lipoprotein (HDL)-cholesterol, triglycerides, and blood sugar levels were determined by using an auto-analyzer (Model 7600-310, Hitachi, Tokyo). Low-density lipoprotein (LDL)-cholesterol levels were calculated according to the Friedewald formula. Fasting plasma insulin concentrations in non-diabetes subjects were measured by enzyme immunoassay (Mercodia Insulin ELISA, Mercodia AB, Uppsala, Sweden). Insulin sensitivity was assessed by the homeostasis model assessment of insulin resistance (HOMA-IR) and β-cell function (HOMA-β), calculated basing on Matthews et al. [21]. As most patients with type 2 diabetes were treated with oral hypoglycemic agents or insulin, these phenotypes were evaluated in non-diabetic controls and diabetic subjects with only diet control to avoid the influence of treatment (n = 513). The body mass index (BMI) was obtained from the ratio of weight (kg) to height squared (m^2^). The estimated glomerular filtration rate (eGFR) was calculated according to the simplified version of the Modification of Diet in Renal Disease Study prediction equation formula, modified by Ma et al. for Chinese patients (eGFR = 175×plasma creatinine^-1.234 ^× age^-0.179 ^× 0.79 [if female]) [22].

### Genotyping

Genomic DNA was extracted from leukocytes by standard procedures using Gentra Purgene Blood Kit (Qiagen, Hilden, Germany). Six tag SNPs in *DDAH1*, rs233112, rs1498373, rs1498374, rs587843, rs1403956, rs1241321 were selected from the HapMap with minor allele frequencies >0.2 and an r^2 ^threshold of 0.8 in the Han population. SNP rs233112 was genotyped by restriction fragment length polymorphism and the others by high-throughput matrix-assisted laser desorption/ionization time-of-flight (MALDI-TOF) mass spectrometry using the SEQUENOM Mass ARRAY system (Sequenom, Sam Diego, CA, USA). The success rate for MALDI-TOF mass spectrometry is 99.7%. All genotypes were determined blinded to the clinical characteristics and 1 negative control and 3 sequenced positive controls were tested on each plate to ensure consistency in genotyping. Primer sequences are available upon request.

### Statistical analysis

Continuous data are presented as the mean ± standard deviation or with a confidence interval (CI) of 95% and differences between groups were compared with one-way analysis of variance (ANOVA) or two-sample *t*-tests. Categorical data were compared by Chi-square test or Fisher's exact test. Univariate logistic/linear regression analysis for the association with type 2 diabetes/log HOMA-IR were tested first for age, gender, hypertension, hypercholesterolemia, smoking, BMI, ADMA, L-arginine/ADMA ratio, eGFR, and genotypes/haplotypes. Those factors with p < 0.1 were included into the multivariate logistic/linear regression analysis. Estimates of pairwise linkage disequilibrium (LD) values (D') were obtained using the HaploView software (version 4.2). Haplotype blocks were estimated using the CI method by Gabriel et al. [23]. Haplotype frequencies were estimated by the GENECOUNTING program (version 1.9). Haplotypes with frequencies greater than 5% were considered in the analysis and the most common haplotype was used as the reference.

Actuarial event-free survival curves were estimated using the Kaplan-Meier method and compared by log-rank test. The univariate Cox regression analysis was performed first to determine the association with the risk of all-cause death and MACE in all subjects, and the variables tested included age, gender, diabetes, hypertension, hypercholesterolemia, smoking, BMI, ADMA, eGFR, and genotypes/haplotypes. Those with a p value < 0.1 were included into the multivariate Cox regression analysis. The plasma ADMA level was tested as a continuous or categorical variable. The hazards ratio (HR) and 95% CI were calculated. A p value of less than 0.05 was considered statistically significant. The SPSS 17.0 (SPSS Inc., Chicago, Illinois) software package was used for statistical analysis.

## Results

Clinical characteristics of cases and controls are summarized in Table 1. In comparison with controls, those with type 2 diabetes had significantly higher BMI, triglyceride, and lower HDL-cholesterol, which are characteristics of type 2 diabetes. In particular, patients with type 2 diabetes had lower eGFR and were more likely to have hypertension. Lower LDL- cholesterol and total cholesterol levels in diabetic patients may be due to their more common use of statins (p = 0.06). The prevalence of hypercholesterolemia was nearly identical in both groups (Table 1).

**Table 1:** Clinical characteristics of type 2 diabetic patients and non-diabetic subjects

	Type 2 DM patients n = 309	Non-DM subjects n = 505	P value
Age (years)	65.4 ± 10.4	64.8 ± 10.8	0.57
Gender (Women/men)	74/235	123/382	0.90
BMI (Kg/m^2^)	27.2 ± 3.9	25.6 ± 3.4	< 0.001
Hypertension (%)	252 (81.0)	348 (68.9)	< 0.001
Smoking (%)	64 (20.7)	106 (21.0)	0.93
Hypercholesterolemia (%)	139 (45.1)	228 (45.1)	1.00
Cholesterol (mg/dl)			
Total	164.7 ± 33.4	175.1 ± 37.3	< 0.001
HDL-Cholesterol	40.1 ± 11.1	44.9 ± 12.7	< 0.001
LDL-Cholesterol	99.6 ± 28.4	110.0 ± 33.6	< 0.001
Triglyceride (mg/dl)	155.0 ± 96.8	138.0 ± 85.4	< 0.001
Creatinine (mg/dl)	1.3 ± 1.0	1.1 ± 0.5	< 0.001
eGFR (ml/min per 1.73 m^2^)	70.8 ± 30.0	80.1 ± 24.9	< 0.001
Fasting blood sugar(mg/dl)	138.0 ± 48.3	92.7 ± 14.5	< 0.001
ADMA (μmol/l)	0.46 ± 0.09	0.45 ± 0.10	0.12
L-arginine (μmol/l)	88.3 ± 29.7	97.3 ± 33.3	< 0.001
L-arginine/ADMA	196.0 ± 67.0	225.0 ± 86.1	< 0.001
*Medications*			
Anti-platelet drug	272 (88.0)	420 (83.2)	0.07
Statins	144 (46.4)	201 (39.8)	0.06
ACE-Inhibitor/ARB	108 (35.0)	111 (22.0)	<0.001
Calcium channel blocker	69 (22.3)	115(22.8)	0.88
Hypoglycemic treatments			
Insulin (%)	49 (15.8)	-	-
OHA (%)	244 (79.0)	-	-
Insulin + OHA (%)	8 (2.6)	-	-

ACE: angiotensin converting enzyme; ARB: angiotensin receptor blocker. BMI: body mass index; eGFR: estimated glomerular filtration rate; OHA: oral hypoglycemic agents.

### Plasma ADMA levels, L-arginine/ADMA ratio and type 2 diabetes

Plasma ADMA levels in all study participants were correlated positively with age (r = 0.20, p < 0.001) and negatively with eGFR (r = -0.21, p < 0.001), but were similar between individuals with and without type 2 diabetes (Table 1). Plasma L-arginine level and the L-arginine/ADMA ratio, an index of L-arginine bioavailability, were both significantly lower in patients with type 2 diabetes (Table 1). By multivariate logistic/linear regression analysis, the L-arginine/ADMA ratio was associated with type 2 diabetes (OR = 0.95/L-arginine/ADMA ratio increase of 10, 95% CI = 0.93-0.97, p < 0.001) and was negatively associated with the log HOMA-IR in non-diabetic subjects (r = -0.157, p < 0.0001), but plasma ADMA was not.

### DDAH1 genotype and type 2 diabetes

**Figure 1:**
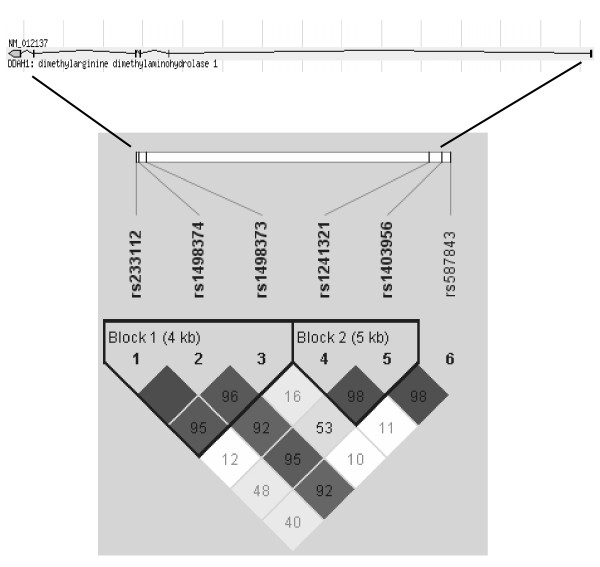
**Linkage disequilibrium plot for 6 SNPs in the DDAH1 gene**. The Haplotype blocks were estimated according to the CI method using the criteria of Gabriel et al (*Science *2002, **296**:2225-2229 [23]).

Genotype distributions for the 6 *DDAH1 *SNPs are summarized in Table 2 and all are in Hardy-Weinberg equilibrium (p > 0.01). SNPs rs1241321-rs1403956 and rs233112-rs1498374-rs1498373 were in tight LD according to D' and belong to 2 haplotype blocks as revealed in the HapMap (Figure 1). We first assessed the association of each *DDAH1 *SNP with type 2 diabetes (Table 2). Among the 6 SNPs, only rs1241321 was significantly associated with the risk of type 2 diabetes in the multivariate logistic regression analysis (A *versus *G: OR = 0.75, 95% CI = 0.60-0.93, p = 0.008 and p = 0.048 when adjusted for multiple comparisons; AA *versus *GG + AG, OR = 0.64, 95% CI = 0.47-0.87, p = 0.004 and p = 0.024 when adjusted for multiple comparisons). The association remained unchanged after adjustment for plasma ADMA levels (OR = 0.64, 95% CI = 0.47-0.86, p = 0.004). Moreover, the fasting plasma glucose and log HOMA-IR in non-diabetic subjects tended to be lower in subjects carrying homozygous AA genotype (Table 3). The baseline clinical characteristics of subjects with AA and GG + AG genotypes are summarized in Table 4 and there were no significant difference between groups except that the subjects with GG + AG genotypes had more cases of type 2 diabetes.

**Table 2:** Association between the DDAH1 gene polymorphisms and type 2 diabetes

SNP ID	Genotype	Type 2 DM patients, n (%)	Non-DM subjects, n (%)	Multivariate adjusted OR* (95% CI), p value
rs233112				
	AA	98 (31.8)	149 (29.6)	
	AG	153 (49.7)	256 (50.9)	0.99 (0.78-1.19)
	GG	57 (18.5)	98 (19.5)	0.72

rs1498374				
	GG	217 (70.2)	347 (69.0)	
	AG	85 (27.5)	146 (29.0)	0.99 (0.74-1.32)
	AA	7 (2.3)	10 (2.0)	0.94

rs1498373				
	CC	138 (44.8)	237 (46.9)	
	CT	137 (44.5)	231 (45.7)	1.21 (0.96-1.52)
	TT	33 (10.7)	37 (7.3)	0.11

rs1241321				
	GG	49 (15.9)	66 (13.1)	
	AG	160 (51.8)	225 (44.6)	0.75 (0.60-0.93)
	AA	100 (32.4)	214 (42.4)	0.008 **

rs1403956				
	GG	180 (58.3)	295 (58.5)	
	AG	118 (38.2)	185 (36.7)	0.99 (0.77-1.27)
	AA	11 (3.6)	24 (4.8)	0.92

rs587843				
	CC	46 (14.9)	87 (17.3)	
	CG	169 (54.7)	239 (47.5)	0.93 (0.75-1.15)
	GG	94 (30.4)	177 (35.2)	0.51

* Odds ratio (OR) for rs233112: A versus G; rs1498374: A versus G; rs1498373: T versus C; rs1241321 A versus G; rs1403956 A versus G; rs587843: G versus C.

** adjusted for multiple comparison: p = 0.048; AA *vs *GG +AG: OR (95% CI) = 0.64 (0.47-0.87), p = 0.004.

**Table 3:** Association between diabetic phenotypes and *DDAH1 *SNP rs 1241321 in non-diabetic subjects

	AA (n = 212)	GG + AG (n = 301)	P value*
Fasting plasma glucose	91.2 (89.6-92.8)	93.7 (91.8-95.6)	0.048
HOMA-IR	3.0 (2.7-3.3)	3.2 (2.9-3.5)	0.15
HOMA-β	47.6 (42.6-52.6)	51.2 (46.8-55.6)	0.18
Log HOMA-IR	0.36 (0.31-0.40)	0.41 (0.38-0.45)	0.015

HOMA-IR: homeostasis model assessment of insulin resistance; HOMA-β: homeostasis model assessment of β-cell function. *: adjusted for age, sex and BMI.

**Table 4:** Clinical characteristics of subjects with rs1241321 AA genotype and GG + AG genotypes

	AA n = 314	GG + AG n = 500	P value
Age (years)	64.3 ± 10.2	65.5 ± 10.2	0.13
Gender (Women/men)	77/237	120/380	0.87
BMI (Kg/m^2^)	26.2 ± 3.8	26.2 ± 3.5	0.98
Diabetes (%)	100 (31.4)	209 (41.8)	0.004
Hypertension (%)	233 (74.2)	367 (73.4)	0.80
Smoking (%)	67 (21.3)	103 (20.6)	0.80
Hypercholesterolemia (%)	155 (49.4)	212 (42.4)	0.05
Cholesterol (mg/dl)			
Total	171.2 ± 35.2	171.2 ± 36.9	0.99
HDL-Cholesterol	43.7 ± 12.3	43.1 ± 12.2	0.49
LDL-Cholesterol	105.5 ± 31.1	106.1 ± 32.7	0.80
Triglyceride (mg/dl)	146.4 ± 88.3	149.8 ± 92.4	0.61
Creatinine (mg/dl)	1.2 ± 0.7	1.2 ± 0.9	0.79
eGFR (ml/min per 1.73 m^2^)	78.0 ± 27.2	75.6 ± 27.3	0.22
Fasting blood sugar(mg/dl)	105.8 ± 35.4	111.7 ± 40.0	0.03
ADMA (μmol/l)	0.46 ± 0.10	0.45 ± 0.10	0.66
L-arginine (μmol/l)	93.1 ± 30.9	94.4 ± 33.0	0.60
L-arginine/ADMA	210.3 ± 76.6	216.3 ± 82.9	0.30
*Medications*			
Anti-platelet drug	269 (85.7)	423 (84.6)	0.68
Statins	129 (41.1)	216 (43.2)	0.55
ACE-Inhibitor/ARB	83 (26.4)	136 (27.2)	0.81
Calcium channel blocker	65 (20.7)	119(23.8)	0.30
Hypoglycemic treatments			
Insulin (%)	19 (6.1)	40 (8.0)	0.30
OHA (%)	81 (25.8)	163 (32.6)	0.04
Insulin + OHA (%)	2 (0.6)	6 (1.2)	0.43

ACE: angiotensin converting enzyme; ARB: angiotensin receptor blocker. BMI: body mass index; eGFR: estimated glomerular filtration rate; OHA: oral hypoglycemic agents.

Next we examined the association between *DDAH1 *polymorphisms and plasma ADMA levels by multivariate linear analysis and found that 4 of the 6 SNPs, except rs1241321 and rs587843, were associated with plasma ADMA levels, with the most significant being the SNP rs1498373 (p = 1.4 × 10^-5^). However, there were no associations between these SNPs and plasma L-arginine levels or L-arginine/ADMA ratio (Table 5).

**Table 5:** Association between the DDAH1 gene polymorphism and plasma ADMA levels, L-arginine levels and L-arginine/ADMA ratio

SNP ID	Genotype	ADMA μmol/l mean (95% CI)	L-arginine μmol/l mean (95% CI)	L-arginine/ADMA mean (95% CI)
rs233112				
	AA	0.45 (0.43-0.46)*	95.8 (91.7-99.9)	222.5 (212.0-233.0)
	AG	0.45 (0.44-0.46)	92.4 (89.2-95.5)	210.7 (202.9-218.5)
	GG	0.47 (0.46-0.49)	95.1 (91.7-96.2)	209.3 (208.5-219.6)

rs1498374				
	GG	0.45 (0.44-0.46)*	93.8 (91.1-96.5)	216.2 (209.5-222.9)
	GA	0.46 (0.45-0.47)	94.0 (89.8-98.2)	210.5 (200.0-220.9)
	AA	0.51 (0.46-0.57)	94.7 (81.5-107.9)	214.0 (208.4-219.5)

rs1498373				
	CC	0.44 (0.43-0.45) ***	94.0 (90.6-97.3)	219.5 (211.0-227.9)
	CT	0.46 (0.45-0.47)	93.3 (90.0-96.5)	210.4 (202.2-218.7)
	TT	0.49 (0.46-0.52)	96.8 (89.2-104.5)	204.0 (187.7-219.6)

rs1241321				
	GG	0.45 (0.43-0.47)	94.8 (88.5-101.0)	220.1(203.5-236.6)
	GA	0.45 (0.44-0.46)	94.2 (90.9-97.5)	215.1 (207.0-223.2)
	AA	0.46 (0.45-0.47)	93.1 (89.7-96.6)	210.3 (201.8-218.8)

rs1403956				
	GG	0.45 (0.44-0.46)*	98.0 (91.1-96.9)	217.6 (210.2-224.9)
	GA	0.46 (0.45-0.47)	93.2 (89.4-96.9)	209.4 (200.3-218.5 )
	AA	0.49 (0.46-0.52)	98.7 (88.4-109.1)	203.3 (182.1-224.6)

rs587843				
	CC	0.46 (0.44-0.48)	92.3 (86.5-98.0)	207.7 (193.6-221.7)
	CG	0.45 (0.44-0.46)	94.3 (91.2-97.2)	216.7 (209.3-224.3)
	GG	0.46 (0.45-0.47)	94.2 (90.2-98.3)	212.8 (202.7-223.0)

*: p < 0.05, ***: p < 0.001, compared by one-way analysis of variance test.

### Clinical outcomes and ADMA/DDAH 1 genotypes

Over a median follow-up period of 28.2 months (25-75% interval: 21.2-46.1 months), there were 44 all-cause deaths (including 24 deaths in type 2 diabetic patients) and 50 MACE (including 31 cardiovascular death, 2 stroke and 17 non-fatal myocardial infarction; including 22 MACE in type 2 diabetic patients). The plasma ADMA level in patients with all-cause mortality and MACE was significantly higher than that in individuals without adverse events (all-cause mortality: 0.53 ± 0.14 μmol/l *versus *0.45 ± 0.10 μmol/l, p < 0.001; MACE: 0.53 ± 0.13 μmol/l *versus *0.45 ± 0.10 μmol/l, p < 0.001). In multivariate Cox regression analysis, the ADMA quartile was identified as an independent predictor of future all-cause mortality (HR = 1.58/ADMA quartile increase, 95% CI = 1.15-2.16; p = 0.004) and MACE (HR = 1.39/ADMA quartile increase, 95% CI = 1.03-1.86; p = 0.03). By considering the plasma ADMA level as a continuous variable, the plasma ADMA level remained a significant independent predictor for all-cause mortality (HR = 1.59/ADMA increase of 0.1 μmol/l, 95% CI = 1.28-1.97; p < 0.001) and MACE (HR = 1.47/ADMA increase of 0.1 μmol/l, 95% CI = 1.18-1.84; p = 0.001). In the multivariate analyses in subgroups of diabetic and non-diabetic subjects, the ADMA quartile remained a significant predictor of all-cause mortality and MACE only in non-diabetic subgroup (non-diabetic group: all-cause mortality: HR = 1.66/ADMA quartile increase, 95% CI = 1.07-2.56; p = 0.02; MACE: HR = 1.94/ADMA quartile increase, 95% CI = 1.22-3.08; p = 0.005; diabetic subgroup: both p = NS; interaction p = 0.04 for all-cause mortality, p = 0.02 for MACE, respectively).

**Figure 2:**
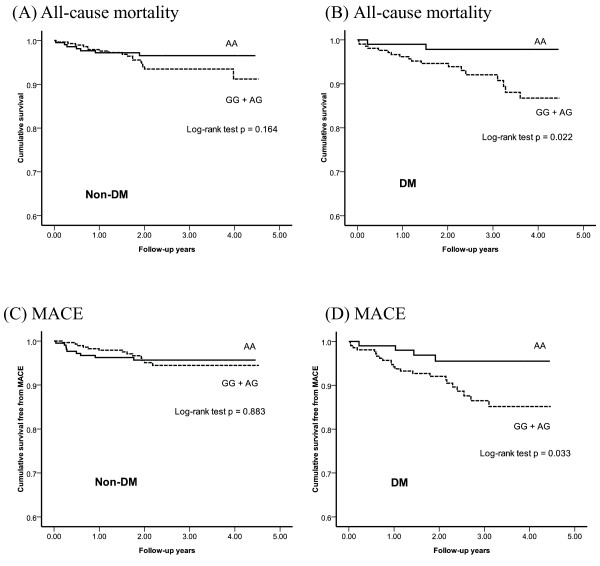
**Kaplan-Meier survival analyses with all-cause mortality (A, B) and major adverse cardiovascular events (MACE) (C, D) during follow-up according to the genotype of rs1241321 (AA___ *vs. *GG + AG....) in diabetic and non-diabetic groups**. P values by log-rank test were shown.

As for the relation between *DDAH1 *genetic variants and long-term outcome, Figure 2 showed the Kaplan-Meier estimates of all-cause mortality and probability of MACE-free survival of patients stratified by genotype of *DDAH1 *rs1241321 in diabetic and non-diabetic groups, and the prognosis of diabetic patients carrying the rs1241321 AA genotype appeared to be better than those carrying GG +AG genotype. Multivariate Cox regression analysis showed that while AA genotype in the *DDAH1 *rs1241321 was not a predictor for long-term outcome in the whole population, it was independently associated with reduced risk for long-term all-cause mortality (AA *versus *GG + AG: HR = 0.18, 95% CI = 0.04-0.80, p = 0.02) and MACE (AA *versus *GG + AG: HR = 0.31, 95% CI = 0.11-0.90, p = 0.03) in type 2 diabetic patients but not in the non-diabetic subgroup (interaction p = 0.16 for all-cause mortality, p = 0.05 for MACE, respectively). There were no significant association between the other SNPs and the long-term clinical outcomes.

### Haplotype-based analysis

Seven common haplotypes with >5% frequency were reconstructed in the study population and accounted for 93.1% of all the estimated haplotypes (Table 6). There was a significant difference in the distribution of haplotype frequencies between subjects with and without type 2 diabetes (permutation test by GENECOUNTING program, p = 0.03). In a multivariate logistic regression analysis adjusted for age, gender, BMI and hypertension, the haplotype H5 (GGCAGC) was found to be significantly associated with a decreased risk of type 2 diabetes (OR = 0.67, 95% CI = 0.46-0.98, p = 0.04, Table 6), which remained unchanged after adjustment for plasma ADMA levels (OR = 0.67, 95% CI = 0.46-0.98, p = 0.04, Table 6). On the other hand, after adjusting for age, gender and eGFR, a significant association with higher plasma ADMA levels was found in haplotypes H3 (GATAAG, p = 4.1 × 10^-4^) and H4 (GGTGGC, p = 2.8 × 10^-4^). There was no significant association between diabetes-related phenotypes and other genotypes or haplotypes. Moreover, no common haplotype was associated with long-term outcomes.

**Table 6:** Association of common haplotypes of DDAH1 gene and type 2 diabetes

Haplotype	Allele						Estimated frequency (%)	OR (95% CI)	P value
				
	rs233112	rs1498374	rs1498373	rs1241321	rs1403956	rs587843			
H1	A	G	C	G	G	G	18.5	-	-
H2	A	G	C	A	G	G	16.0	0.76 (0.53-1.08)	0.13
H3	G	A	T	A	A	G	15.2	0.95 (0.67-1.36)	0.79
H4	G	G	T	G	G	C	13.7	1.18 (0.83-1.69)	0.36
H5	G	G	C	A	G	C	13.2	0.67 (0.46-0.98)	0.04
H6	A	G	C	A	G	C	9.8	0.97 (0.66-1.44)	0.89
H7	A	G	C	A	A	G	6.7	0.88 (0.55-1.40)	0.58

## Discussion

In this study, SNP rs1241321 in *DDAH1 *was found to be associated with a higher risk of type 2 diabetes independently of the plasma ADMA level. In addition, individuals with an AA genotype at rs1241321 appeared to be more insulin-sensitive when compared with AG/GG individuals. Over a median follow-up period of 28.2 months, AA genotype at rs1241321 was associated with better long-term clinical outcome in diabetic subgroup. In contrast, some SNPs of *DDAH1*, especially the rs1498373, might influence the plasma level of ADMA. However, with the exception of rs1241321, none of these SNPs or the plasma ADMA level was associated with type 2 diabetes, suggesting that the interaction of *DDAH1 *variants with type 2 diabetes may not be directly related to its enzymatic activity, i.e. not just simply mediated by the plasma ADMA level. We also identified a common haplotype H5 (GGCAGC) that was associated with reduced risk of type 2 diabetes.

It is well recognized that type 2 diabetes and its metabolic derangements such as hyperinsulinemia, hyperglycemia, dyslipidemia, and increased oxidative stress are associated with NO-mediated endothelial dysfunction [1]. In contrast, endothelial dysfunction may lead to attenuated glucose uptake in insulin-sensitive tissues, hyperglycemia, and ultimately to the development of insulin resistance and type 2 diabetes [2,3]. Furthermore, impaired NO bioavailability plays a pivotal role in the regulation of glucose-stressed endothelial progenitor cell dysfunction in type 2 diabetes, and anti-oxidant treatment with superoxide dismutase may restore their function [24]. ADMA, an endogenous competitive inhibitor of NO synthase, is known to impair NO bioavailability and endothelial function [4-7]. Elevated ADMA level might inhibit the mobilization, differentiation and function of endothelial progenitor cell [25]. Therefore, accumulating evidences suggested that ADMA might be implicated in the pathogenesis of insulin resistance [8,26] and the vasculopathy in type 2 diabetes. As human plasma ADMA is eliminated primarily by the DDAH enzymes [15], it has been demonstrated that both heterozygous *DDAH1 *knock-out mice and vascular endothelial-specific *DDAH1*-deficient mice showed significantly increased ADMA levels and endothelial dysfunction [27,28]. In addition, over-expression of *DDAH1 *led to reduced plasma ADMA level and enhanced insulin sensitivity [17,29]. Wolf et al. recently reported that reduced urinary ADMA concentrations were associated with impaired cardiac function and might predict future cardiovascular risk. Moreover, they found that the urinary dimethylamine (the metabolic breakdown product of ADMA by DDAH)/ADMA ratio was significantly increased in patients with severe coronary artery disease (CAD), suggesting that DDAH might be up-regulated in CAD [30]. However, the influence of *DDAH1 *polymorphisms on plasma ADMA levels in human is rarely addressed. Recently, Abhary et al. reported that the genetic variation in *DDAH1 *was significantly associated with serum ADMA levels in subjects with type 2 diabetes [31]. They tested 26 tag SNPs in *DDAH1*, including rs1241321, rs587843, and rs1498373 that we tested in the present study. Similar to our results, they showed that, among the 26 tag SNPs, rs1498373 was significantly associated with serum ADMA level, while rs1241321 and rs587843 were not. Nevertheless, since all these SNPs are located in the intron regions of *DDAH1*, their functional significances and the underlying mechanisms by which these polymorphisms affect the serum ADMA levels remain unclear. In contrast, in the present study it was intriguing to find that SNP rs1241321, which did not affect the plasma ADMA level, was associated with insulin sensitivity, the risk of type 2 diabetes and their long-term outcome. Furthermore, adjustment for the plasma ADMA level did not attenuate its effect on the risk of type 2 diabetes or its prognostic value, suggesting that its role on type 2 diabetes is not likely to be mediated through plasma ADMA. To the best of our knowledge, the present study is the first one to investigate the association of *DDAH1 *polymorphism with type 2 diabetes and to assess the prognostic significance of *DDAH1 *polymorphism in these patients. However, the functional significance of SNP rs1241321 remains unclear. SNP rs1241321 is located in intron 1 of *DDAH1 *(Chr1: 85,927,629, GRCh37), about 3 Kb from the novel *DDAH1 *c.-397_ -396insGCGT polymorphism (Chr1: 85,931,124, GRCh37), which was recently reported to disrupt the binding of transcription factors and resulted in significantly reduced *DDAH1 *transcription and elevated plasma ADMA level [32]. Nevertheless, the correlation between these two polymorphisms may not be high, because of the relatively large discrepancy in their minor allele frequencies (0.377 for rs1241321, and 0.10 for c.-397_ -396insGCGT in Chinese Han Denver). Caplin et al. reported that the *DDAH1 *SNP rs233112 that we tested was significantly associated with plasma ADMA level in several cohorts of chronic kidney disease [33]. Intriguingly, they found that another *DDAH1 *SNP rs17384213 GG genotype was associated with lower plasma ADMA level, increased expression of DDAH1 mRNA in kidney allograft, but in contrast was associated with more rapid decline of renal function. The latter observation was conflicting with a previous observational study [34]. Similarly, the functional significances of these *DDAH1 *SNPs were also unknown. Although possible mechanisms include effects on local tissue or the intracellular concentration of ADMA, the exact functional role of SNP rs1241321 and other *DDAH1 *SNPs requires detailed investigated in further studies.

Several studies have shown that elevated plasma ADMA levels were present in patients with types 1 and 2 diabetes with retinopathy/nephropathy [10-12], and is usually associated with a worse long-term cardiovascular prognosis in diabetic patients [13,14]. However, in the present study, the plasma ADMA level did not relate to the risk of type 2 diabetes, but the L-arginine/ADMA ratio did. This may be due to different ethnicity or clinical characteristics of the study population, as our population was composed of subjects referred for diagnostic coronary angiography, and had a higher prevalence of concomitant CAD and atherosclerosis risk factors such as hypertension and hypercholesterolemia. In a large-scale study of 3238 individuals scheduled for coronary angiography, the plasma ADMA levels of patients with type 2 diabetes were only marginally higher than those of the patients without diabetes (0.83 μmol/l *versus *0.82 μmol/l, p = 0.032) [35]. In another large community-based population study, the mean plasma ADMA level was similar between non-diabetic and diabetic subjects (0.546 μmol/l *versus *0.553 μmol/l, p = NS). Furthermore, in this study type 2 diabetes was a significant independent predictor of plasma concentrations of L-arginine and L-arginine/ADMA ratio, but not plasma ADMA level [36], which is similar to our results. Interestingly, Sibal et al. reported recently that the plasma ADMA levels in patients with early type 1 diabetes without macrovascular disease or macroalbuminuria were even significantly lower compared to healthy controls. In addition, the plasma ADMA levels were not associated with impaired flow-mediated dilatation of brachial arteries in these diabetic patients [37]. Therefore, these conflicting evidences suggest that the association of ADMA and type 2 diabetes is elusive. Since it has been shown that increased NO synthase-derived free radical production may be one of the resources of oxidative stress in diabetes [38,39], one might speculate that the inhibition of uncoupled endothelial NO synthase by ADMA with resulting paradoxical reduction of oxidative stress might be a possible explanation for the conflicting relation of ADMA with cardiovascular events in patients with uncomplicated diabetes [40]. In summary, these findings suggest that the interactions among plasma ADMA level, *DDAH1 *polymorphism, and type 2 diabetes are much more complex than previously thought and are far from being completely understood. Thus, further investigations for the relationship between ADMA and type 2 diabetes are warranted.

There are several limitations in this study. First, our study subjects were enrolled from individuals referred for coronary angiography, and with higher prevalence of CAD and risk factors of atherosclerosis. Confirmation in a larger cohort of Chinese Han or a Caucasian population and those with less co-morbidity may be mandatory. Second, the information about complications of type 2 diabetes, including microalbuminemia, nephropathy and retinopathy, was unavailable in this study. Considering the close association of plasma ADMA with these diabetic complications in previous reports, studies investigating their association with the *DDAH1 *polymorphisms may be informative. Finally, it has been reported that several pharmacological agents might lower the plasma ADMA level, especially angiotensin converting enzyme inhibitor/angiotensin receptor blocker and statins [41,42], which were used more commonly in our diabetic group. However, there were no significant differences in plasma ADMA level between patients using angiotensin converting enzyme inhibitor/angiotensin receptor blocker (p = 0.59) and statins (p = 0.14) or not.

## Conclusion

Our study provides the first evidence that SNP rs1241321 in *DDAH1 *is associated with insulin sensitivity, type 2 diabetes and their long-term prognosis, independently of plasma ADMA levels. The *DDAH1 *gene may play a potential role in the pathogenesis of type 2 diabetes.

## Abbreviations

ADMA: asymmetric dimethylarginine; BMI: body mass index; CAD: coronary artery disease; DDAH: Dimethylarginine dimethylaminohydrolase; eGFR: estimated glomerular filtration rate; HOMA-IR: homeostasis model assessment of insulin resistance; MACE: major adverse cardiovascular events; NO: nitric oxide; SNP: single nucleotide polymorphisms.

## Competing interests

The authors declare that they have no competing interests.

## Authors' contributions

TML, SJL and MYC: conceived the study, obtaining funding, writing the draft and revising the manuscript; TML: collecting and analyzing data; CPH and MWL: statistical analysis and discussion of results. All authors read and approved the final manuscript.
